# Animal Experiments in Biomedical Research: A Historical Perspective

**DOI:** 10.3390/ani3010238

**Published:** 2013-03-19

**Authors:** Nuno Henrique Franco

**Affiliations:** Institute for Molecular and Cell Biology, University of Porto, Rua do Campo Alegre, 823, 4150-180 Porto, Portugal; E-Mail: nfranco@ibmc.up.pt; Tel.: +351-226-074-900

**Keywords:** animal research, animal testing, biomedical research, animal ethics, history of science

## Abstract

**Simple Summary:**

This article reviews the use of non-human animals in biomedical research from a historical viewpoint, providing an insight into the most relevant social and moral issues on this topic across time, as well as to how the current paradigm for ethically and publically acceptable use of animals in biomedicine has been achieved.

**Abstract:**

The use of non-human animals in biomedical research has given important contributions to the medical progress achieved in our day, but it has also been a cause of heated public, scientific and philosophical discussion for hundreds of years. This review, with a mainly European outlook, addresses the history of animal use in biomedical research, some of its main protagonists and antagonists, and its effect on society from Antiquity to the present day, while providing a historical context with which to understand how we have arrived at the current paradigm regarding the ethical treatment of animals in research.

## 1. Introduction

Animal experimentation has played a central role in biomedical research throughout history. For centuries, however, it has also been an issue of heated public and philosophical discussion. While there are numerous historical overviews of animal research in certain fields or time periods, and some on its ethical controversy, there is presently no comprehensive review article on animal research, the social controversy surrounding it, and the emergence of different moral perspectives on animals within a historical context. This perspective of animal use in the life sciences and its moral and social implications from a historical viewpoint is important to gauge the key issues at stake and to evaluate present principles and practices in animal research. 

This review aims to provide a starting point for students and scholars—either in the life sciences or the humanities—with an interest in animal research, animal ethics, and the history of science and medicine. The reader interested in a more in-depth analysis on some of the topics reviewed is referred to the reference list for suggestions of further reading.

## 2. From Antiquity to the Renaissance

Humans have been using other vertebrate animal species (referred to henceforth as animals) as models of their anatomy and physiology since the dawn of medicine. Because of the taboos regarding the dissection of humans, physicians in ancient Greece dissected animals for anatomical studies [1]. Prominent physicians from this period who performed “vivisections” (*stricto sensu* the exploratory surgery of live animals, and historically used *lato sensu* as a depreciative way of referring to animal experiments) include Alcmaeon of Croton (6th–5th century BCE) [2,3], Aristotle, Diocles, Praxagoras (4th century BCE), Erasistratus, and Herophilus (4th–3rd century BCE) [1,3,4]. The latter two were Hellenic Alexandrians who disregarded the established taboos and went on to perform dissection and vivisection on convicted criminals, benefiting from the favorable intellectual and scientific environment in Alexandria at the time [1]. All of these authors had a great influence on Galen of Pergamon (2nd–3rd century CE), the prolific Roman physician of Greek ethnicity who developed, to an unprecedented level, the techniques for dissection and vivisection of animals [3,5] and on which he based his many treatises of medicine. These remained canonical, authoritative, and undisputed until the Renaissance [1,6].

For most ancient Greeks, using live animals in experiments did not raise any relevant moral questions. The supposed likeliness of humans to their anthropomorphic deities granted them a higher ranking in the *scala naturae* (“the chain of being”), a strict hierarchy where all living and non-living natural things—from minerals to the gods—were ranked according to their proximity to the divine. This view of humans as superior would later influence and underline the Judeo-Christian perspective of human dominion over all nature, as represented by texts by Augustine of Hippo (IV century) and Thomas Aquinas (XIII Century), the most influential Christian theologians of the Middle Ages. For Augustine, animals were part of a natural world created to serve humans (as much as the “earth, water and sky”) and humankind did not have any obligations to them. For Thomas Aquinas, the mistreatment of another person’s animal would be sinful, not for the sake of the animal in itself, but because it is someone else’s property. Cruelty to animals was nevertheless condemned by Aquinas, as it could lead humans to develop feelings and actions of cruelty towards other humans. Also, for this theologian, one could love irrational creatures for the sake of charity, the love of God and the benefit of fellow humans (for selected texts, see reference [7]). 

The belief amongst ancient Greek physicians that nature could be understood by means of exploration and experiment—and the medical knowledge thus obtained to be of clinical relevance in practice—would be replaced by other schools of medical thought. Most notably, the Empiric school (3rd century BCE–4th century) would reject the study of anatomy and physiology by dissection of cadavers or by vivisection, not only on the grounds of cruelty and the established taboos, but also for its uselessness. Empiricists believed pain and death would distort the normal appearance of internal organs and criticized the speculative nature of the conclusions drawn from experiments. Indeed, and despite taking an experimental approach to understand the human body and illness, the interpretations of physiological processes made by ancient Greeks who performed vivisections were often inaccurate. The theoretical frameworks by which physicians interpreted their experiments more often than not led them to misguided conclusions. Observations would be understood in light of such paradigms as the Hippocratic theory of the four humors or the Pythagorean theory of the four elements, along with others of natural or supernatural basis, and to which they added their own theoretical conceptions and observational errors [1,4,6,8,9]. The study of human or animal anatomy and physiology was hence deemed irrelevant for clinical practice. Beginning with the decline of the Roman Empire and continuing throughout the Middle Ages, physiological experiments—along with scientific activity in general—would fall almost entirely into disuse and medical knowledge would become dogmatic. In an increasingly Christianized Europe, there was little motivation to pursue scientific advancement of medical knowledge, as people became more concerned with eternal life than with worldly life, and returned to Pre-Hippocratic beliefs in supernatural causes for disease and in the healing power of faith and superstition. Therefore, and despite medieval physicians’ reverence for Galen and his predecessors, the experimental approach used by these classical authors had been sentenced to oblivion [3,8,9,10,11]. 

The use of animal experiments to satisfy scientific enquiry would only re-emerge in the Renaissance. Flemish anatomist Vesalius (1514–1564), through the course of his work as a physician and surgeon, realized that many anatomical structures thought to exist in humans—on account of them being present in other animals—were in fact absent [6]. This led him to break the established civil and religious rules and dissect illegally obtained human cadavers, and publish very accurate descriptions of the human anatomy, which challenged the authority of the classical authors. As Herophilus did centuries before (but not carried on by his successors) [1] Vesalius would also examine the similarities and differences between the internal structure of humans and other animals, thus setting the foundations of modern comparative anatomy. 

Alongside the progress in anatomical knowledge made possible by experimenters defying the Catholic Church’s opposition to the dissection of human bodies, the Renaissance period also witnessed the resurgence of vivisection as a heuristic method for the understanding of animal physiology. Vesalius would again recognize the value of physiological experiments on animals as both a learning and teaching resource—he would vivisect animals for medical students as the finishing touch at the end of his courses—a view shared by his contemporary, and presumable student and rival, Realdo Colombo (1516–1559) [3]. Later, Francis Bacon (1561–1626), considered by many the founder of modern scientific methodology, would also approve of the scientific relevance of vivisection, stating that “the inhumanity of *anatomia vivorum* was by Celsus justly reproved; yet in regard of the great use of this observation, the inquiry needed (…) might have been well diverted upon the dissection of beasts alive, which notwithstanding the dissimilitude of their parts may sufficiently satisfy this inquiry” [12].

## 3. Seventeenth Century and the Dawn of the Enlightenment

Physiological experiments on animals carried on throughout the seventeenth century, in the period favorable to scientific progress now known as the Age of Enlightenment. René Descartes’s (1596–1650) description of animals as “machine-like” [13] was heavily criticized by many of his contemporaries, but nevertheless provided scientists a way to justify what would now be considered extremely gruesome experiments [3,14,15,16] in a time when anesthesia, for humans and animals alike, was not available. It has been argued, however, that Descartes’s views on animals were misinterpreted [17,18]—misconstructions that may not always have been free from malice, either by his contemporaries [19] or present-day critics [20]—as he did not explicitly state that animals were incapable of feeling pain and indeed recognized them to be able to do so insofar as it depends on a bodily organ, and even admitted animals to be capable of such sentiments as fear, anger, hope or joy [13]. Nonetheless, regardless of it being misinterpreted or not (for a discussion see [21]), Cartesian *machinism* would be recurrently evoked in defense of vivisection in the 17th and 18th centuries [14,15,16]. Malebranche, following his interpretation of Descartes, would explicitly justify vivisection on the grounds of it only being “apparently harmful” to animals [3,15,22]. Also, as someone deeply interested in physiology and medicine [23,24], and a “man of his time,” Descartes performed vivisections himself [15,16,21], an activity for which his—perhaps more apologetic than wholehearted—view of animals as soulless, senseless *automata *“absolved man from the suspicion of crime” [25]. 

**Figure 1:**
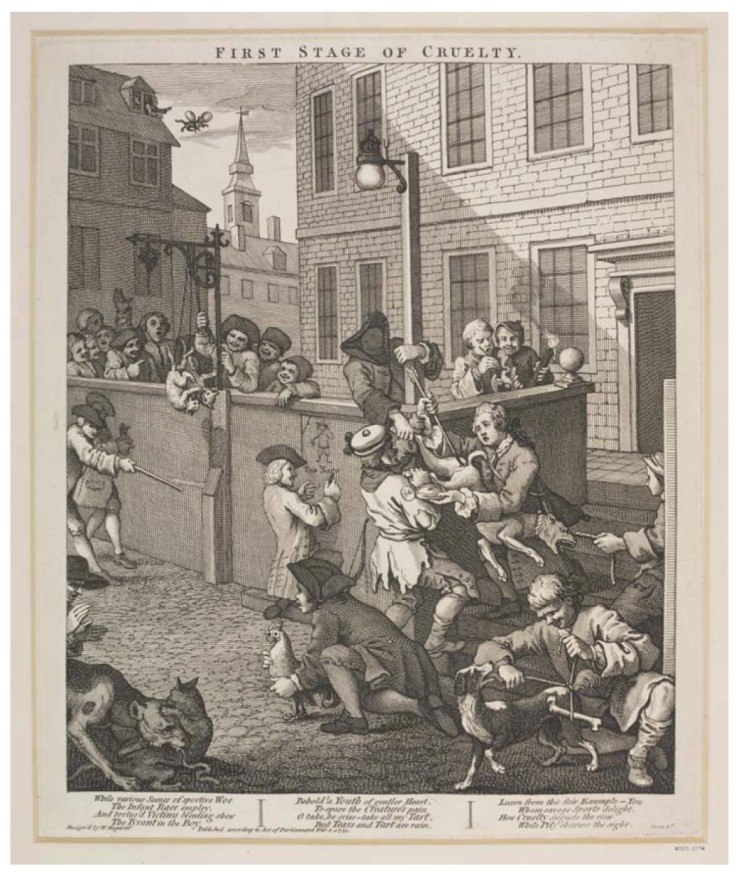
“First Stage of Cruelty” by William Hogarth (1750), the first plate from “The Four Stages of Cruelty” series, which describes the escalating violent behavior that follows childhood cruelty to animals to an adulthood of criminal life. In this scene, two boys plunge an arrow into the rectum of a dog, while another boy, most likely the pet’s owner, pleads with them to stop. Meanwhile, some boys are burning the eye of a bird, while others tie bones to a dog’s tail. Also, some boys play “cock-throwing” (a popular sport in eighteenth-century England, consisting of throwing stones or bottles at a cockerel tied to a stake) while others hang fighting cats, and others even throw animals from windows. Source: ^©^Victoria and Albert Museum, London.

As for other contemporary philosophers, Baruch Spinoza (1632–1677) did not deny animals’ ability to feel, but considered we should nevertheless “use them as we please, treating them in a way which best suits us; for their nature is not like ours” [26], whereas John Locke (1632–1704) fully recognized that animals could feel and stated that children should be brought up to abhor the killing or torturing of any living thing in order to prevent them from later becoming capable of cruel actions to fellow humans [27]. Immanuel Kant (1724–1804) would reject Cartesian mechanistic views, thus acknowledging sentience to other animals. However, Kant would not extend his concept of human intrinsic and inalienable dignity to other species. In his *Of Duties to Animals and Spirits*, and mirroring Thomas Aquinas’s views on the subject, he observed that “all animals exist only as means, and not for their own sakes, in that they have no self-consciousness, whereas man is the end (…) it follows that we have no immediate duties to animals; our duties towards them are indirect duties to humanity” [28]. Kant believed his anthropocentric philosophy provided the moral tradition and contemporary thought of his society; it was a philosophical underpinning, rather than an abstraction distant from the thoughts and feelings of the ordinary man [29]. Indeed, his argument that cruelty against animals would lead to cruelty to humans was—as it continues to be—popular amongst the public and scholars (e.g., [30]). In *Duties to Animals, *Kant would refer to William Hogarth’s (1697–1764) popular series “The Four Stages of Cruelty” (Figure 1), a set of four engravings that depicted how cruel actions against animals could lead to moral degradation and crime. Regarding animal use in research, Kant would state that “Vivisectionists, who use living animals for their experiments, certainly act cruelly, although their aim is praiseworthy, and they can justify their cruelty, since animals must be regarded as man’s instruments; but any such cruelty for sport cannot be justified” [28]. While he believed actions that offended human intrinsic dignity were unacceptable—no matter how laudable their ultimate purpose should be—when it came to animals it would not be the actions themselves, but rather their justification that defined the acceptability of those actions. While the Enlightenment marked the beginning of the departure from Christian theocentrism, in the new anthropocentric view, animals continued to have no moral standing on their own. In perspective, it should be noted this was a time in which the slave market thrived and women were seen as inferior. However, the recognition of animals’ sentience in the new philosophical thought would later be instrumental for new ethical perspectives to arise on the moral status of animals. 

Amidst the list of notable Western seventeenth-century physiologists using animals, the most noteworthy was undoubtedly William Harvey (1578–1657), physician to kings James I and Charles I, and one of the founders of modern science. In 1628, his groundbreaking *Exercitatio Anatomica de Motu Cordis et Sanguinis in Animalibus* (“An Anatomical Exercise on the Motion of the Heart and Blood in Living Beings”) was published, in which he provided the most accurate description of blood circulation and heart function of his time [31,32,33,34]. Using the results of meticulously planned experiments on live animals, as well as their interpretation through mathematics and physics, in this treatise, Harvey disproved many of Galen’s fifteen-hundred-year-old ideas [35,36]. In the tradition of his own academic lineage (he studied in Parma with the renowned anatomist Fabricius, a pupil of Colombo), Harvey was also a prolific and skilled comparative anatomist, whose studies on the anatomy of animals included species of several taxa, including mammals, fish, amphibians, reptiles and even insects [37].

Harvey’s *De Motu Cordis* was highly criticized, since his experimental observations did not fit the prevalent theories of Western natural philosophy of his time (for an insight on the social, scientific and academic context surrounding Harvey see [33,37,38]), still heavily grounded on Galenic principles. Harvey’s findings would challenge firmly established beliefs, such as blood being continuously produced in the liver and transported through the veins to be consumed by other organs, while arteries were thought to be filled with air; the heart was believed to have a heating—rather than pumping—function, and blood was thought to flow between the ventricles across a permeable septum; the vascular system as a whole was thought to be open; the arterial and venous bloods were believed not to mix; and the mere concept of blood circulation was virtually unknown (however, his teacher Fabricius might already have envisaged the concept of blood circulation [34]. Also, blood circulation was already known in Chinese medicine sixteen centuries before Harvey [39]). From an epistemological point of view, such opposition also reflected a dispute between the empiricist and the rationalist approach to the understanding of nature, for Harvey professed “to learn and teach anatomy not from books but from dissection, not from the tenets of Philosophers but from the fabric of Nature” (from *De Motus Cordi*, cited in [32]). Not surprisingly, Descartes—although a researcher himself—disagreed emphatically with most of Harvey’s findings, since he believed that theories forged through philosophical reflection on metaphysics were superior to those resulting from experimental observation, thus considering experiments or interpretations that did not confirm his own natural philosophy as flawed [40,41]. He nevertheless praised Harvey’s discovery of circulation and the method of experiment and observation that had led to it, a support that would actually help to turn the tide amidst scholars in favor of Harvey’s observation-over-doctrine ideas and methodological approach on experimental physiology, thereby setting the ground for further developments in physiological knowledge [38].

Further advancements in physiology would be prompted by questions left unsolved by Harvey, many of them addressed by an ensemble of his colleagues and followers at Oxford who applied Harvey’s principle that life should be interpreted in light of new findings in physics in their physiological experiments on animals [42,43,44,45]. The *Oxford Group* included polymaths like Robert Hooke (1635–1703), John Locke (1632–1704), John Mayou (1640–1679), Richard Lower (1631–1691), Thomas Willis (1621–1675), Robert Boyle (1627–1691) and Christopher Wren (1632–1723), amidst several others. Most physiologists did not expect direct therapeutic applications to result from their experiments [45]. There were, however, a few exceptions, such as Lower’s attempts at intra and inter-species blood transfusions having in mind their medical application, or Johann Wepfer’s (1620–1695) use of animals as a proxy to humans to infer the toxicity of several substances [3], a practice that is still carried out to this day. Seventeenth-century physiology would mark the dawn of modern scientific inquiry in the life sciences. Animal experiments were now proving to be more informative and relevant for obtaining scientifically sound knowledge on basic biological processes than ever before. These advancements would eventually diminish the importance of Galenic dogmatic medicine—although some of its principles would still endure for many years—and ultimately pave the way for today’s evidence-based medicine. 

The seventeenth century would also witness the advent of skepticism towards experiments on animals on scientific grounds. Physicians like Jean Riolan, Jr. (1580–1657) and Edmund O’Meara (1614–1681) began to question the validity of physiological experiments carried out on animals in such an extremely altered state as one endured under vivisection, although their hidden agenda was to restore the credibility of Galenic medicine [3,46,47]. This dispute between critics and advocates of the informative value of animal models of human physiology still echoes today, e.g., [48].

**Figure 2:**
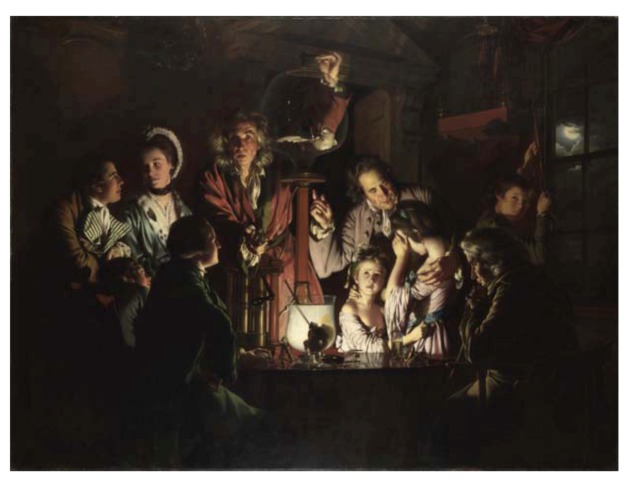
“An Experiment on a Bird in an air pump”, by Joseph Wright of Derby (detail) (1768). In this brilliant artwork, the artist captures the multiple reactions elicited by the use of live animals as experimental subjects in eighteenth-century Britain, for which we can find a parallel in present day’s diverse attitudes on this topic, including shock, sadness, appreciation, curiosity and indifference. Currently in *The National Gallery*, London. Source: *Wikimedia Commons*.

The moral acceptability of inducing suffering in animals on the physiologist’s workbench would also become an issue raised in opposition of vivisection before the end of the seventeenth century [3]. However, the acceptance of the animal-machine paradigm by many physiologists reassured them that their scientific undertakings were not cruel. Furthermore, even the many who acknowledged that animals suffered a great deal with experiments, nevertheless defended themselves against the accusation of cruelty by alleging that the suffering inflicted was not unjustified, but rather for the sake of humankind, in the same line of reasoning by which today animal research is still justified. Nevertheless, these scientists were often overwhelmed by the extreme ill treatment they forced themselves to carry out on fully conscious animals [3,45,49]. One such investigator was Robert Boyle, whose infamous experiments on live animals on an air pump (conceived by him and developed by Robert Hooke) consisted in registering how animals responded to increasingly rarefied air. While only two animal experiments in Boyle’s “pneumatic chamber” are described in his *New Experiments Physico-Mechanical Touching the Spring of the Air and its Effects* (1660)—he would nevertheless go on to publish further animal studies on physiology [50,51]. Public demonstrations of this experiment would become very popular in the eighteenth century, although it bore more of an entertaining, rather than educational, nature (Figure 2). 

## 4. Eighteenth Century and the Rise of Moral Consideration for Animals

Amongst the many remarkable physiologists of the eighteenth century, polymaths Stephen Hales (1677–1761) and Albrecht von Haller (1708–1777) stood out. Hales was responsible for the first measurement of pressure in the blood vessels, and for other important insights into cardiovascular and respiratory physiology [52,53,54]. He also gave landmark contributions to public health and other medical breakthroughs, including the invention of forceps. Von Haller was arguably the most prolific physiologist of his time, better known for his groundbreaking work on inflammation, neurophysiology, heart function, and hemodynamics [55,56,57,58,59,60]. Both researchers were disgusted by the gruesomeness of their own experiments and were concerned about their moral justification, but nevertheless carried on, certain of the need for the use of live animals for the comprehension of many basic physiological processes, which were yet far from being understood [3,49,61,62]. Other relevant landmarks of eighteenth-century biomedical science based on animal studies included the foundation of experimental pharmacology [63], electrophysiology [64,65], and modern embryology [66]. Despite these advancements in biological knowledge, the clinical relevance of animal studies continued to be challenged [3,61,62] and, indeed, direct benefits to human health from animal experiments would remain elusive throughout the eighteenth century [45,55] and well into the following century. 

Opposition to vivisection had raised its tone since the beginning of the eighteenth century, prompted by the popularization of public displays of experiments on live animals—in particular the notorious demonstrations of Boyle’s notorious air pump experiments [3,61,62], which were seen as purposeless, and thus inherently cruel—but became more prominent in the second half of the century, particularly in northern Europe [3,61,62,67]. Anthropocentric views on human duties to animals began to become increasingly challenged by philosophers, from Voltaire’s (1694–1778) criticism of Cartesian machinism and the gruesomeness of animal experiments [68] to Jean-Jacques Rousseau’s (1712–1778), Jeremy Bentham’s (1748–1832) and Arthur Schopenhauer’s (1788–1860) criticism of those who viewed animals as mere “means to an end.” By referring to sentience rather than intelligence to grant animals inherent worth, these philosophers proposed a shift from an anthropocentric justification for our duties of kindness to animals, to human obligations towards other animals for the sake of the animals themselves [69,70,71]. Rousseau proposed that despite animals being unable to understand the concept of natural law or rights, they should nonetheless, as a “consequence of the sensibility with which they are endowed (…) partake of natural right.” While Bentham found the concept of natural right “nonsense” [72], he sanctioned the idea of granting animals moral standing for the sake of their sentience. As he would famously state: “The question is not, Can they reason? Nor, Can they talk? But, Can they suffer?” [71]. From his utilitarian philosophy standpoint (*i.e.*, that a moral action is that which results in the highest overall wellbeing for all stakeholders), he deemed animal research acceptable, provided the experiment had “a determinate object, beneficial to mankind, accompanied with a fair prospect of the accomplishment of it,” thus admitting that humans had precedence over other animals, limited by the due consideration for their suffering [73]. Bentham’s utilitarianism continues to exert a great deal of influence in today’s debate on animal use in the life sciences.

Among philosophers and physiologists alike, the issue of discussion was now not if animals could feel or not and to what extent, but rather whether vivisection was justifiable based on the benefit for human beings derived from it. Thus, even when researchers had strong misgivings about the inflicted suffering of animals, benefit to humans remained a valid justification for them to pursue their scientific goals through vivisection [61]. While knowledge of bodily functions and pathology was still incipient at that time, eighteen-century physiologists differed from their seventeenth-century predecessors, as they believed that medical improvements could one day be achieved through advancing knowledge by the means of animal experimentation [62]. The same rationale—that human interests took precedence over animal suffering—would also be used by nineteenth-century physicians as an ethical justification for the use of animals. 

## 5. The Nineteenth-Century Medical Revolution and the Upsurge of the Antivivisection Societies

By the beginning of the nineteenth century, medicine was undergoing a major revolution. The organization of medical practice was changing, with the construction of hospitals, the university training of medical doctors, and the invention of new instruments and methods for the medical profession [74]. There was also a growing acknowledgement by the medical community that most medical practice, up to that period, was based on unproven traditions and beliefs and that most therapies were not only ineffective but often worsened the patient’s condition. As a result, medical practice increasingly began to focus more on understanding pathology and disease progression, pursuing more accurate diagnosis and prognosis, and thus providing reliable and useful information to patients and families, as they realized this was often the best they could do at the time. This paradigm shift would help give more credit and recognition to medical doctors and scientists, who, at that time, were often viewed with disdain and suspicion by the general public. This gain in medical knowledge would, however, sometimes be at the expense of unapproved trials, invasive procedures, and no respect for what we would now call patients’ rights [75,76,77]. 

**Figure 3:**
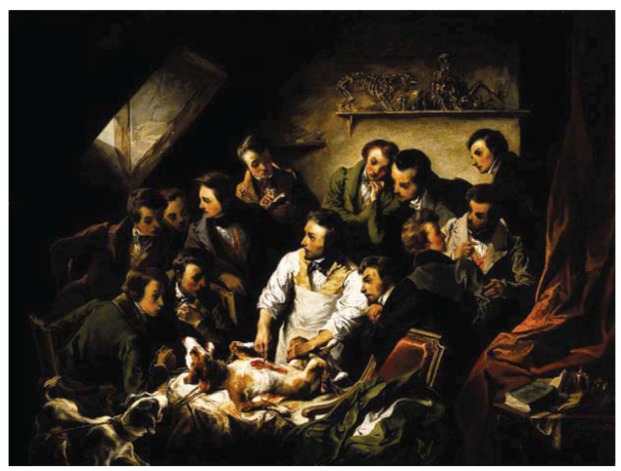
“A physiological demonstration with vivisection of a dog,” by Émile-Édouard Mouchy. This 1832 oil painting—the only secular painting known of the artist—illustrates how French scholars valued physiological experimentation in service of scientific progress [90]. Notice how the struggling of the animal does not seem to affect the physiologist or his observers. Currently part of the *Wellcome Gallery* collection, London. Source: *Wellcome Library*.

Another kind of medical revolution was taking place in the laboratories, one that would ultimately provide the consistent basic science on which twentieth-century modern medicine would set its foundations. This scientific revolution began with a political one. The French Revolution of the late eighteenth century would later, in the first-half of nineteenth century, set the grounds for the establishment of the *Académie Royale de Médecine*, a thriving academic environment where science—and physiology, chemistry, and pharmacy, in particular—would finally be incorporated into medicine. The acknowledgment of the great knowledge gap in physiology and pathology, and the openness to positivist views on scientific knowledge, led to the definitive abandonment of the quasi-esoteric and, up to that time, dominant vitalistic theories in physiology, which stated that a vital principle, the “soul”, was the main source of living functions in organisms, rather than biochemical reactions. This led to a generalization of the understanding of all bodily processes as an expression of physical and chemical factors, and to a greater relevance given to animal experiments for answering scientific questions (Figure 3). At the *Académie*, animal experiments were being increasingly prompted by existing clinical problems, and carried out with the ultimate goal of developing new therapeutic approaches to tackle these issues. Importantly, the integration of veterinarians in the *Académie* was deemed valuable for their insight on such experiments [57,78,79]. Amidst many other prominent scientists, two physician–physiologists stood out for their contributions to experimental physiology, François Magendie (1783–1855) and, most notably, Magendie’s disciple, Claude Bernard (1813–1878) [67,80,81,82,83,84]. Bernard’s experimental epistemology, unlike his tutor’s more exploratory approach, advocated that only properly controlled and rigorously conducted animal experiments could provide reliable information on physiology and pathology of medical relevance, setting the landmark of experimental medicine [85,86,87,88]. Conciliating Descartes’s rationalism with Harvey’s empiricism, Bernard acknowledged the importance of ideas and theories for the formulation of hypotheses, safeguarding, however, that these were only useful if testable and only credible if substantiated through experimentation [80,89]. He seemed to have been aware of how important and groundbreaking his approach to medical knowledge would become, when in his opening remarks to medical students in his very first lecture he quoted himself from his seminal “Introduction to the Study of Experimental Medicine,” stating: *The scientific medicine that I’m responsible to teach does not yet exist. We can only prepare the materials for future generations by founding and developing the experimental physiology which will form the basis of experimental medicine”* [89]. 

From the 1830s and throughout the second half of the century, the concept of scientific medicine would also flourish amidst a distinct group of German/Prussian physiologists. Following the rationale that biology could be understood through the means of chemistry and physics, and through their pivotal animal experiments and the use of microscopy, these scientists vastly contributed to the development of anatomy, histology, pathology, embryology, neurophysiology, physiology and physics. The setting for this scientific and epistemological progress was the *Anatomisches Museum* in Berlin, where anatomist, zoologist, and physiologist Johannes Müller (1801–1858) offered workspace and supervision to brilliant students whose independent research he wished to encourage. Although lacking the money, space, and instruments available in the great German laboratories founded after 1850, the museum provided these young scientists—notably Theodor Schwann (1810–1882), Robert Remak (1815–1865), and Friedrich Henle (1809–1885) in the 1830s, and Carl Ludwig (1816–1895), Emil du Bois-Reymond (1818–1896), Ernst Brücke (1819–1892), Hermann von Helmholtz (1821–1894), and Rudolph Virchow (1821–1902) in the 1840s—a singular intellectual atmosphere for research. Henle and Virchow would become leaders of the 1840s’ medical revolution in Germany, promoting the reform of medicine by providing it with a scientific basis, while Brücke, Helmholtz, and Bois-Raymond’s focused on the development of physiology as an autonomous science [83,91,92,93,94,95]. Their contributions to medical knowledge through the nineteenth century, along with Magendie’s and Bernard’s pivotal works, would deeply influence their counterparts across the Western world in the latter decades of the nineteenth century. Thousands of students flocked to attend medical schools in Germanic universities (and French institutes, although to a lesser extent), many of them from across the Atlantic [85,88,91,96,97]. This, in turn, would lead to an unprecedented rise in animal research-based advancement in biological and medical knowledge in the late nineteenth century—with important consequences for public health and quality of life—as further discussed later in this text.

While the second half of the nineteenth century marked the beginning of scientifically meaningful and medically relevant animal research, this period also saw opposition to vivisection becoming a more widespread idea in Europe, especially in Britain. Although animal experiments were not yet regulated in the first half of the century, the development of British physiology research in the Victorian Era was losing pace to Germany and France, where unprecedented progress in medical knowledge was taking place. The openly antivivisectionist positions of influential jurists, politicians, literary figures, clergymen, distinguished members of the medical community, and even Queen Victoria, contributed to an unfriendly environment for animal-based medical research [90,91]. There was, however, also a matter of divergence of opinion between British anatomists and French physiologists on which was the best approach for obtaining medical knowledge. Taking advantage of the rising antivivisection trend, British anatomists explored the (undoubted) gruesomeness of Magendie’s experiments, along with some nationalistic partisanship and xenophobic feelings against France, in their defense of anatomical observation as the primary method for advancing physiology, to the detriment of experiment through vivisection. However, they seldom disclosed their own positive (or at least ambivalent) views on animal experiments as a means to corroborate findings achieved through anatomical exploration [90,98,99]. Magendie would become the arch-villain of the antivivisection movement. Despite the broad recognition of his contributions to science by most peers, he was also amongst the most infamous of his time for the disdain he held for his experimental subjects. This contestation was louder outside of France, where many of his fellow scientists, even those who approved of animal experimentation, described him as an exceptionally cruel person who submitted animals to needless torture [85,90,100]. His public presentations became the most notorious, particularly one he performed in England when he dissected a dog’s facial nerves while the animal was nailed down by each paw, and was left overnight for further dissection the following day [82]. A description of Magendie’s classes to medical students by an American physician added further to the widespread disgust directed towards his work:

*This* *surgeon’s spring course of experimental physiology commenced in the beginning of April. I seldom fail of “assisting” at his murders. At his first lecture, a basketful of live rabbits, 8 glass receivers full of frogs, two pigeons, an owl, several tortoises and a pup were the victims ready to lay down their lives for the good of science! His discourse was to explain the function of the fifth pair of nerves. The facility was very striking with which the professor could cut the nerve at its origin, by introducing a sharp instrument through the cranium, immediately behind and below the eye. M. Magendie drew the attention of the class to several rabbits in which the fifth pair of nerves had been divided several days before. They were all blind of one eye, a deposition of lymph having taken place in the comes, from inflammation of the eye always following the operation alluded to, although the eye is by this section deprived of all its sensibility. Monsieur M. has not only lost all feeling for the victims he tortures, but he really likes his business. When the animal squeaks a little, the operator grins; when loud screams are uttered, he sometimes laughs outright. The professor has a most mild, gentle and amiable expression of countenance, and is in the habit of smoothing, fondling and patting his victim whilst occupied with preliminary remarks, and the rabbit either looks him in the face or ‘licks the hand just raised to shed his blood. During another lecture, in demonstrating the functions of the motive and sensitive fibers of the spinal nerves, he laid bare the spinal cord in a young pup, and cut one bundle after another of nerves. (…) Living dissection is as effectual a mode of teaching as it is revolting, and in many cases the experiments are unnecessarily cruel and too frequently reiterated; but so long as the thing is going on, I shall not fail to profit by it, although I never wish to see such experiments repeated.*

*cit in* Olmsted, 1944 [101]

All of Magendie’s experiments were carried out without anesthesia or analgesia (and animals would be left in agony for hours, or for students’ “hands-on” anatomical studies. While, in fairness, it should be recognized that anesthetics had not yet been discovered when Magendie performed the bulk of his work, even after this technique had become available, he and nearly all of his students continued to forgo anesthesia in their experiments [102]. Moreover, animal studies on the effects of anesthetics themselves (Bernard was responsible for significant contributions to the understanding of the physiology of anesthesia: for an overview, see references [103,104]) were performed, as well as anatomical studies that could well have been conducted with cadavers, with no need for animals to be exposed to such prolonged suffering. Magendie was so ill famed in Britain that his experiments were referenced in the House of Commons by Richard Martin (1754–1834) when he presented a bill for the abolition of bear-baiting and that would become the “Cruel Treatment of Cattle Act” of 1822, one of the first animal protection laws. He would be again evoked in the report favoring the regulation of animal experiments that led to the “Cruelty to Animals Act” of 1876, the first piece of legislation ever to regulate animal experiments. By that time, Magendie had been dead for over twenty years [82,90,100]. 

After Magendie’s death, the focus of antivivisectionists’ attention moved to Bernard’s works, which included cutting open conscious animals under the paralyzing effects of *curare*, or slowly “cooking” animals in ovens for his studies on thermoregulation [105]. Bernard’s line of work would eventually have a heavy personal cost. Tired of her husband’s atrocious experiments, his wife would divorce him—taking with her his two daughters, who grew up to hate him—and, joining the antivivisectionists’ ranks, set up rescue shelters for dogs. Even Bernard’s cause of death is attributed to years of work in a humid, cramped, and poorly ventilated laboratory. He would, however, die a national hero, being given the first state funeral ever to be granted to a scientist in France. In his later years, he would collect the highest academic and political honors, including a seat in the French senate [88,102,106,107]. 

Despite their utter disregard for animal suffering, Magendie and Bernard did not see themselves as the immoral senseless villains portrayed by their detractors, but rather as humanists. Indeed, their view that animals did not deserve the same moral consideration as humans made them condemn experiments in humans without previous work on animals, the general principle on which the use of animal models in biomedical science is still grounded. In a time when proper dosage, administration, and monitoring of anesthesia were still largely unknown, often leading to serious side effects and accidental deaths, Magendie would state, on the use of anesthetics in humans without previous and thorough tests on animals: “That is what I do not find moral, since we do not have the right to experiment on our fellows” [5,108,109]. The amorality of human experiments prior to animal testing in animals was also an ethical argument raised in favor of vivisection by Bernard [89], who wrote:

*No* *hesitation is possible, the science of life can be established only by experiment, and we can save living beings from death only by sacrificing others. Experiments must be made either on man or on animals. Now I think physicians already make too many dangerous experiments on man, before carefully studying them on animals. I do not admit that it is moral to try more or less dangerous or active remedies on patients, without first experimenting with them on dogs; for I shall prove, further on, that results obtained on animals may all be conclusive for man when we know how to experiment properly. If it is immoral, then, to make an experiment on man when it is dangerous to him, even though the result may be useful to others, it is essentially moral to do experiments on an animal, even though painful and dangerous to him, if they may be useful to man.*

British physiologists often refrained from experimenting on mammals, mostly on account of the public’s opposition to the gruesomeness of continental physiologists’ experiments. However, with the publication of Bernard’s book (1868) and John Burdon-Sanderson’s *Handbook for the Physiological Laboratory* (1873), the scientific relevance of animal experiments became increasingly acknowledged, providing a utilitarian justification for vivisection, despite the harm endured by animals, eventually resulting in the rise in animal studies in medical schools in Britain in the 1870s [5,88,99]. Furthermore, by this time, anesthetics were already available and used by British physiologists, leading RSPCA secretary John Colam to state that “laboratory practices in England were very different indeed from [those] of foreign physiologists.” While the usefulness of anesthetics to chemically restrain animals was certainly advantageous for researchers, pain relief was most likely the major reason behind their ready adoption by many physiologists in Britain, as the paralyzing properties of *curare* were already known and used for this purpose. In fact, even before the solidifying of the antivivisectionist struggle, British physiologists had set themselves guidelines for responsible research [110,111]. Nevertheless, many researchers still found the analgesic and anesthetic effect of these volatile agents to be a source of undesired variability, thus avoiding their use altogether [99,105]. 

The upsurge of animal research in Britain was accompanied by an intensification of the antivivisectionist struggle. In 1875, the first animal protection society with the specific aim of abolishing animal experiments was founded: the *Victoria Street Society for the Protection of Animals Liable to Vivisection *(later known as the National Anti-Vivisection Society), led by Irish feminist, suffragist, and animal advocate Frances Power Cobbe (1822–1904). Vivisection became a matter of public debate, only matched in Great Britain that century by the controversy around the 1859 publication of Charles Darwin’s (1809–1882) *On the Origin of Species*, in which he presented a strong scientific rationale for the acknowledgement of our close kinship with the rest of the animal world, giving both physiologists and antivivisectionists a strong argument for their cause, depending on the perspective. 

As the original argument of antivivisectionists that animal research was inacceptable because it did not provide useful medical knowledge began to lose strength (however, it remained a recurrent accusation against animal research, see, for instance, [112]), the discussion shifted towards preventing unnecessary harm, rather than questioning the scientific value of animal experiments [99]. On the other hand, the use of anesthetics now allowed British scientists to argue that most physiological experiments involved little, if any, pain [105,110,113]. While this made some antivivisectionists ponder about their own standing on the use of animals in research—namely those who opposed vivisection on the grounds that the intense and prolonged suffering endured by animals on the physiologist table was intolerable—many others felt that the most relevant value at stake was the preservation of each animal life in itself, questioning if human benefit was sufficient reason for sacrificing animals [99,110]. Moreover, the claim that animals were rendered senseless to pain gave *carte blanche* to many physiologists to use as many animals as they pleased for research, teaching, and demonstrations, despite anesthesia often being improperly administered, thus failing to prevent suffering for more than the brief initial moments. A famous quote by George Hoggan (a former vivisectionist who was appalled to witness Bernard’s experiments and who would later co-found the *Victoria Street Society*) illustrates the relevance of the new ethical issues that emerged: “I am inclined to look upon anaesthetics as the greatest curse to vivisectible animals” [5,99]. 

In the last decades of the nineteenth century, all of today’s most relevant arguments on the debate surrounding the use of animals for scientific purposes were already in place, as well as most of the rhetoric and means of action in defense of each position. These views included outright abolitionism and, on the opposite pole, scientists demanding to be allowed to work without restrictions; non-scientists accusing researchers to be self-biased and unable to think ethically about their work and, on the other side of the barricade, researchers disdaining the authority of non-scientists to criticize their work; the benefit for humankind argument *vs*. the questioning of the scientific and medical value of animal research on scientific grounds; public demand for stronger regulation *vs*. researchers’ appeals for more autonomy, freedom, and public trust; advocates of the justifiability of only applied research (but not basic research) *vs.* apologists of the value of all scientific knowledge, see [105,112,113]. 

Just like today, there were also those who valued both animal protection and scientific progress and, recognizing that each side had both relevant and fallacious arguments, found themselves in the middle-ground, where they sought ways for compromise and progress. Amongst these, the most notable was Charles Darwin, known for his affection to animals and abhorrence for any kind of cruelty, but also for his commitment to scientific reasoning and progress [111,114,115]. Additionally, Joseph Lister (1827–1912), one of the most influential physicians of his time, would decline a request by Queen Victoria in 1875 for him to speak out against vivisection. Lister was one of the few British surgeons that carried out vivisection, albeit only occasionally, and was acquainted with some of the most eminent continental physiologists. In his response letter to the Queen, he pointed out the importance of animal experiments for the advancement of medical knowledge, stressed that anesthetics should be used at all times, and also denounced the ill treatment of animals in sports, cruel training methods, and artificial fattening of animals for human consumption as being more cruel than their use in research [116]. 

With the controversy assuming growing complexity and relevance, two opposing bills were presented to the British parliament in 1875: the “Henniker bill,” named after its sponsor Lord Henniker and promoted by Frances Cobbe, and the “Playfair bill,” named after scientist and Member of Parliament, Lyon Playfair, and promoted by Charles Darwin himself, along with fellow scientists and friends. Despite coming from opposite ends , both bills proposed reasonable regulation of animal experiments, rather than demanding severe restriction or granting scientists unlimited rights to use animals. Somewhat surprisingly, the Playfair bill drafted by researchers was, in some aspects, more restrictive than Henniker’s by proposing, for instance, that animal experiments should only be performed for the advancement of physiology and not for teaching purposes. The crucial difference was that the Henniker bill called for all researchers and all kinds of experiments to be properly licensed and supervised, as it is today in Great Britain, while the Playfair bill proposed that the law should only be applied to painful experiments. In the absence of parliamentary consensus for either one or the other bill, a Royal Commission—properly balanced to include members of the RSPCA and a few eminent scientists, including T.H. Huxley—was appointed that same year to address this issue, which would result in the 1876 amendment of the 1835 Cruelty to Animals Act in order to regulate the use of animals for scientific purposes, being the first case of this kind of legislation in the world [99,111,117,118]. This bill would endure for 110 years, until the enactment of the 1986 Animals (Scientific Procedures) Act, and remain the only known legislation to regulate animal experiments for nearly 50 years, despite some attempts to pass similar bills in other Western countries, where antivivisectionism was growing, particularly in Germany, Switzerland, Sweden, and North America [14,119].

The recrudescence and spread of antivivisection feelings in the late nineteenth century was coincidental with the long-awaited beginning of direct clinical and public health benefit from animal research. Before the end of the century, the germ theory of infectious diseases—*i.e.*, that pathogenic microbes were the causative agent of such diseases, rather than internal causes, “miasmas” in the air or water, or even more esoteric explanations—would gain broad recognition by the medical community, mostly on account of the work of Louis Pasteur (1822–1895) and his German counterpart, Robert Koch (1843–1910), which was largely based on animal experimentation. This knowledge would have an immediate, profound, and enduring effect on public health, surgery and medicine. Although it had been earlier proposed by Ignaz P. Semmelweis (1818–1865) that puerperal fever was caused by infections resulting from poor hygiene of physicians [120], only after Joseph Lister’s paper *On the Antiseptic Principle of the Practice of Surgery* (1867)—prompted by Pasteur’s findings—was the importance of hand-washing and instrument sterilization before surgical procedures and child delivery finally acknowledged, leading to a drastic drop in deaths from puerperal fever and post-surgical sepsis. Until then, previous efforts to make hand-washing a standard procedure had been ridiculed by the medical class. 

Pasteur, a professor of chemistry with a doctoral thesis on crystallography, would turn his attention to biology in 1848 [121]. He began by unraveling the biological nature of fermentation (the inhibiting effect of oxygen on fermentation is still called the “Pasteur effect”), moving on to devise solutions of great economic value by tackling wine and beer spoiling, as well as silkworm disease, all of which he properly identified as being caused by microbes. Together with Claude Bernard, a close friend, he would later develop the process of *pasteurization* to destroy microorganisms in food. Pasteur began hypothesizing that microbes could also be the causative agents of many diseases affecting humans and other animals. Together with his disciples, most notably Emile Roux (1853–1933), he would go on to identify *Staphylococcus*, *Streptococcus*, the “septic vibrio” (now *Clostridium septicum*), the causative agents of anthrax (*Bacillus anthracis*) and chicken cholera (*Pasteurella multocida*), being the first to develop vaccines for these zoonotic diseases, as well as for Swine Erypselas, thus setting the foundations of modern immunology [122]. However, it would be Pasteur’s successful use of a therapeutic vaccine against rabies in humans that would grant him international celebrity status [107,122,123,124]. 

**Figure 4:**
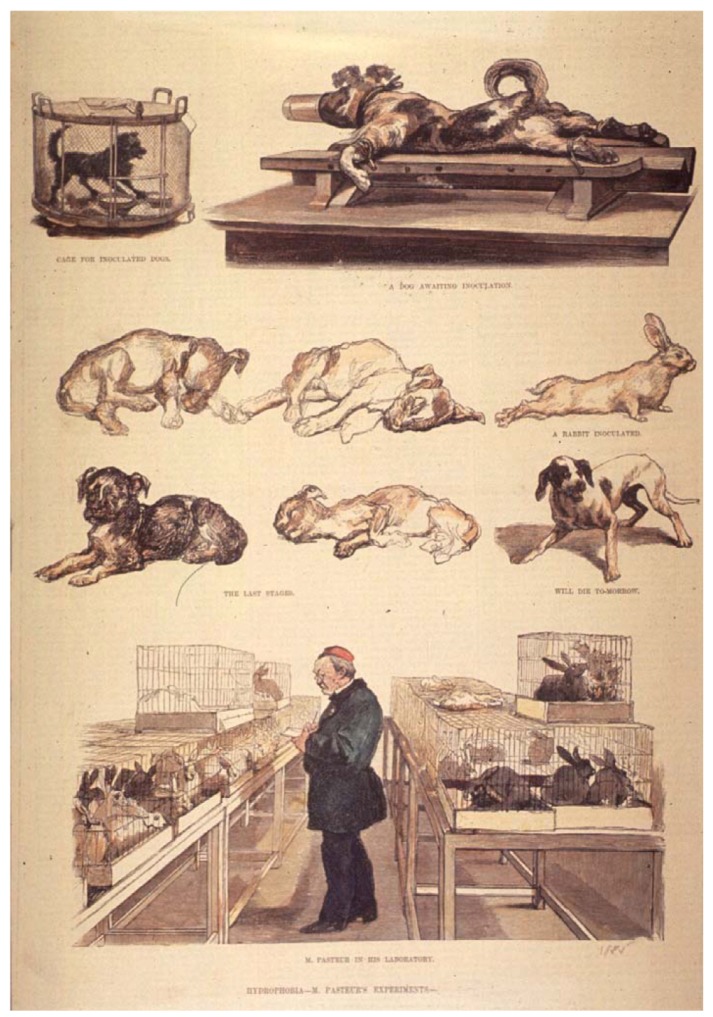
This full-page illustration of Pasteur in his animal facility was published in *Harper’s Weekly* in the United States, on 21 June 1884. At this time, there was moderate curiosity on Pasteur’s work in the US, which would intensify after his first successful human trials of a therapeutic vaccine for rabies in 1885. In the article, the reader is reassured that the use of dogs is both humane and justified in the interest of mankind. The use of other species, however, is barely mentioned [5]. Source: *Images from the History of Medicine*, U.S. National Library of Science.

Pasteur’s work required the experimental infection of numerous animals, as well as inflicting surgical wounds to test antiseptic techniques and disinfectant products, which made him a prime target of antivivisectionists. Either by genuine conviction or pragmatic convenience, amongst the ranks of Pasteur’s critics for his use of animals, one could easily find opponents of vaccination and the germ theory. Pasteur would frequently receive hate letters and threats, mostly for his infection studies on dogs, although he also used chickens, rabbits, rodents, pigs, cows, sheep, and non-human primates (Figure 4). Pasteur was, however, more sensitive to animal suffering than most of his French counterparts. Not only was he uneasy with the experiments conducted—although sure of their necessity—he would also always insist animals be anesthetized whenever possible to prevent unnecessary suffering. He would even use what we now call “humane endpoints” (for a definition, see [125]): in a detailed description of his method for the prophylactic treatment of rabies (from 1884), the protocol for infecting rabbits with the rabies virus (for ulterior extraction of the spinal cord to produce a vaccine), he stated that: “The rabbit should begin to show symptoms on the sixth or seventh day, and die on the ninth or tenth. Usually the rabbit is not allowed to die, but is chloroformed on the last day in order to avoid terminal infections and unnecessary suffering” [126]. Furthermore, he would become directly responsible for saving countless animals from the burden of disease and subsequent culling [5,107,113,127,128].

Robert Koch, a practicing rural physician, would follow the tradition of the great German/Prussian physiologists of his time (and indeed was a student to many of them), providing invaluable contributions to medical knowledge through animal research, mainly in the field of bacteriology and pathology. His famous “Koch postulates” would play an important role in microbiology Along with his associates, Koch developed from scratch methods that are still used today, such as microphotography of organisms, solid medium culture, and staining or microbe quantification. They would go on to identify the causative agents of tuberculosis (*Micobacterium tuberculosis*, also known as the “Koch bacillus”), cholera (*Vibrio cholera*, albeit 30 years after Filippo Pacini, 1812–1883 [129]), and anthrax. The overlapping interest of Pasteur and Koch on anthrax would trigger a bitter rivalry between the two, fuelled by their different approaches to microbiology, as well as chauvinistic Germany–France rivalry [130,131]. Koch’s own school of microbiology housed many of the leading late-nineteenth, early-twentieth-century medical researchers. This included Emil von Behring (1854–1917) and Paul Ehrlich (1854–1915), both responsible for the first antitoxin for treatment of diphtheria—developed from horse serum—for which von Behring received the Nobel Prize in 1901. Von Behring would also develop an antitoxin for immunization against tetanus, along with Shibasaburo Kitasato (1853–1931), who had also studied under Koch. In 1908, Ehrlich would also be awarded the Nobel Prize for contributions to immunology, and would yet again be nominated for his contributions to chemotherapy and the development of Salvasaran (an effective treatment against syphilis), in particular [132,133,134,135]. The development and production of vaccines and antitoxins led to a dramatic increase in the number of animals used in research. The number of animals used by physiologists in the nineteenth century would be negligible in comparison with the several hundred used by Pasteur to develop, test, and produce vaccines, the thousands of mice used by Paul Ehrlich for the production of Salvasaran—his syphilis drug—and the millions of primates that would be used to produce Polio vaccines in the 1950s [5].

## 6. The Twentieth-Century Triumph of Science-Based Medicine

By the end of the nineteenth and beginning of the twentieth century, the pharmacopeia had effective, scientifically tested drugs, a landmark that allowed for an increasing number of people to understand the importance and validity of scientifically sound medical knowledge and, with it, the relevance of animal-based research (see [113,136,137,138]). One could still find as far as the end of the nineteenth century, however, physicians who disregarded the ideals of scientific medicine and vigorously stood by their traditional epistemological view of medicine and clinical practice, which they saw as more of a form of art than as a science. Many such physicians also opposed experiments on live animals and were members of antivivisection societies [77,139,140,141]. Nonetheless, the medical profession, medicine itself, and human health had now been irreversibly changed by science, and would continue to be pushed forward throughout the twentieth century to now. 

The twentieth century would witness astonishing advances in medical knowledge and the treatment of disease. The discovery of vitamins, hormones, antibiotics, safe blood transfusion, new and safer vaccines, insulin, hemodialysis, chemo and radiotherapy for cancer, the eradication of small pox (and the near eradication of poliomyelitis), advanced means of diagnostic and new surgical techniques are but a very few examples of twentieth century’s medical achievements that have not only saved millions of lives—human and non-human—but also allowed countless humans and animals to live a “life worth living,” by the relief of disease-induced suffering. The advances of biomedical research to human health since the dawn of the past century are countless, with animal research playing a role in a number of important discoveries (for an overview, see [142]). Of the 103 Nobel Prizes in physiology or medicine given since 1901, on 83 occasions work conducted on vertebrate species (other than human) was awarded, while in another four instances, research relied heavily on results obtained from animal experiments in vertebrates conducted by other groups [143]. Another indirect measure of the impact that biomedical progress had on the twentieth century was the increase in life expectancy, which in some developed countries doubled between 1900 and 2000, and is still on the rise today [144,145,146]. 

By the 1910–1920s, antivivisection groups were fighting an increasingly difficult war for the public’s support. The argument that no medical progress could be obtained through animal research was becoming increasingly difficult to uphold and, as researchers pledged to avoid animal suffering whenever possible, criticism of animal experiments on the grounds of cruelty toned down. However, not all scientists had sufficient, if any, consideration for animal suffering, and research would continue to be unregulated in most countries. Nevertheless, the exaggerated claims, radical abolitionist views, and scientific denialism by more inflexible antivivisectionists would make them lose support from the general public and more moderate animal protection groups, leading to a decline—albeit not an end—to the antivivisection movement, until its resurgence in the 1970s. Confronted with a general lack of support, moreover in a period that would witness two great world wars and a serious economic recession—which would push the interests of animals further to the background—the line of action of antivivisectionists through most of the twentieth century focused on banning the use of dogs and other companion animals [5,147,148,149,150]. 

The toning down of the opposition to animal use in the life sciences had also something to do with the emergence of rodent species as a recurrent animal model in research. Unlike dogs or horses, rodents like mice and rats were seen as despicable creatures by most of the public, and therefore less worthy of moral consideration, which in turn deemed their use in research more acceptable [147]. While this came as an advantage to researchers, it is hard to say, however, if the actual weight of the public’s misgivings about the use of domestic animals was a relevant contributing factor for the ready adoption of rodent models, especially when considering their other numerous advantages as experimental animals when compared to other species. Firstly, they are small, easy to handle, and relatively cheap to house. Secondly, they are highly resistant to successive inbreeding and have a short lifespan and rapid reproduction rate [151,152]. 

**Figure 5:**
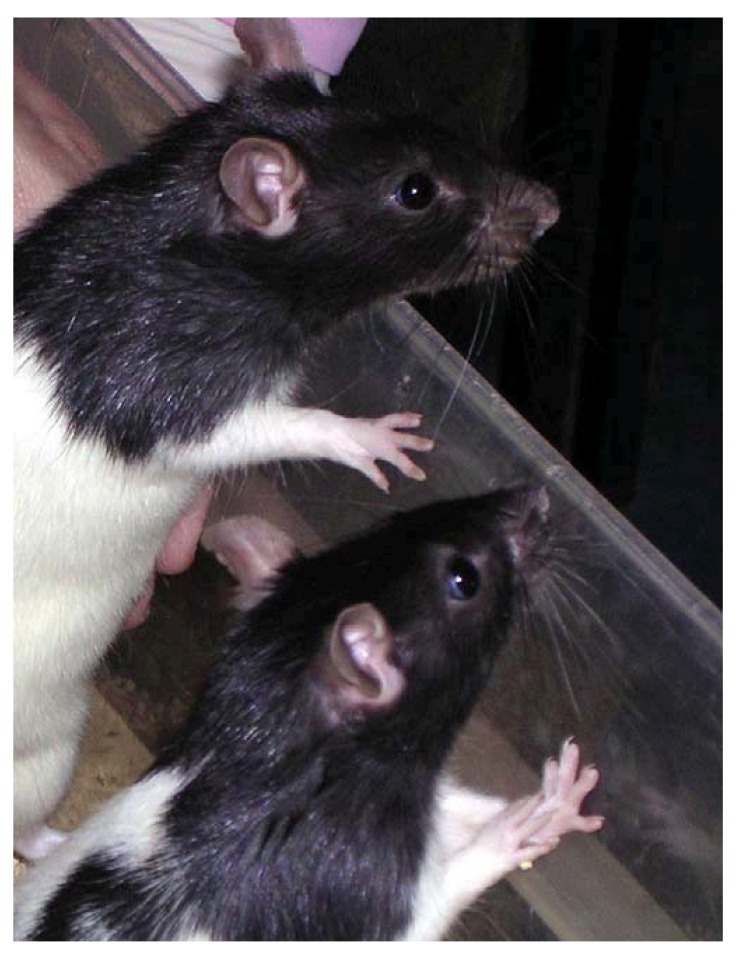
Two outbred laboratory rats, of the Lister Hooded (Long–Evans) strain. Rodents are the most commonly used laboratory animals, making up nearly 80% of the total of animals used in the European Union, followed by cold-blooded animals (fish, amphibian and reptiles, making up a total of 9.6%) and birds (6.3%) [159] Photo: Francis Brosseron, reproduced with permission.

Domesticated rats (*Rattus norvegicus*) were the first rodent species to be used for scientific purposes. Their use in physiological research dates to as early as 1828, but only in the first decades of the twentieth century did they become a preferred tool in research, after the development in 1909 of the first standard rat strain, the *Wistar Rat*, from which half of all rats used in laboratories today are estimated to have descended (for a historical perspective, see [153,154]) (Figure 5). The mouse (*Mus musculus*) had also been used in the nineteenth century, famously by Gregor Mendel in his 1850s studies on heredity of coat color, until the local bishop censored mouse rearing as inappropriate for a priest, which made him turn to using peas instead [155]. The mouse would be again picked up in the beginning of the nineteenth century by Lucien Cuénot (1866–1951) to demonstrate that mammals also possessed “genes” (a vague concept at the time) that followed the laws of Mendelian inheritance, and would since then become a privileged model in the study of genetics, a field that would grow exponentially after the discovery of the DNA structure in 1953 by James Watson (born 1928) and Francis Crick (1916–2004). In 1980 John Gordon and Franck Ruddle developed the first transgenic mouse [156], and in 1988, the first gene knockout model was produced, which granted Mario R. Capecchi (born 1937), Martin J. Evans (born 1941), and Oliver Smithies (born 1925) the 2007 Nobel Prize. In 2002, the mouse became the second mammal, after humans, to have its whole genome sequenced. These, along with other technologies, have opened unlimited possibilities for the understanding of gene function and their influence in several genetic and non-genetic diseases, and have made the mouse the most commonly used animal model in our day (for a historical overview of the use of the mouse model in research, see [157,158]), with prospects being that it will continue to have a central role in biomedicine in the foreseeable future. 

## 7. Animal Liberation and the Pathway for a More Humane Science

Opposition to animal experiments resurged in the second half of the twentieth century, in particular after the 1975 publication of *Animal Liberation* by Australian philosopher Peter Singer (born 1946) [160]. Singer offered a strong philosophical grounding for the animal rights movement, by arguing that the use of animals in research—as well as for food, clothing or any other purpose—is mostly based on the principle of *speciesism* (coined by Richard Ryder in 1970 [161]), under which animals are attributed a lower moral value on the sole basis of belonging to a different species [162], which he considers to be no less justifiable than racism or sexism. His argument, however, does not stem from the premise that animals have intrinsic rights. As a preference utilitarian—and unlike hedonistic utilitarians like Bentham and Mill who argued we should act in order to maximize net happiness—Singer proposed that our actions should aim to do what on balance “furthers the interests of those affected” [163]. Holding that the interest of all sentient beings to both avoid pain and have positive experiences deserves equal consideration, he thus argues that it is difficult to justify animal research, since it generally does not hold to Bentham’s “*Each to count for one and none for more than one*” postulate. Furthermore, it is usually unfeasible to prospectively quantify how many may benefit directly from a given animal experiment. According to Singer, by using the principle of equal consideration of interests, one should give priority to relieving the greater suffering. Singer does not propose we should assume different species suffer similarly under the same conditions but, on the contrary, that care should be taken when comparing the interests of different species as, for instance, a human cancer patient, for his higher cognitive skills, can suffer a great deal more than a mouse with the same disease [164]. For this reason, he does not consider animal research to always be morally wrong in principle, and even admits that in certain occasions it may be justifiable, albeit these situations are, in his view, exceptional [165]. 

The animal rights movement would, however, receive from American philosopher Tom Regan (born 1938) a more uncompromising view of our duties to animals than Singer’s utilitarianism, one that would question the use of animals in research—or in any other way—altogether, regardless of the purpose of research. In Regan’s book *The Case for Animal Rights* (1983), he proposed we should extend the Kantian concept of intrinsic value to all sentient beings. This perspective inherently affords vertebrates rights, despite their incapacity to understand or demand such rights, as it is also the case—Regan argues—of small infants and the severely mentally handicapped. Hence, the respect for the life and wellbeing of sentient animals should be taken as absolute moral values, which can only be violated in very specific and extreme cases—such as self-defense. Regan’s moral philosophy hence only allows for an abolitionist view on animal research—since no “ends” can justify the “evil means” of sacrificing an animal in the face of the inviolable dignity of sentient beings [166]—and has become the main theoretical reference for the animal rights movement. 

**Figure 6:**
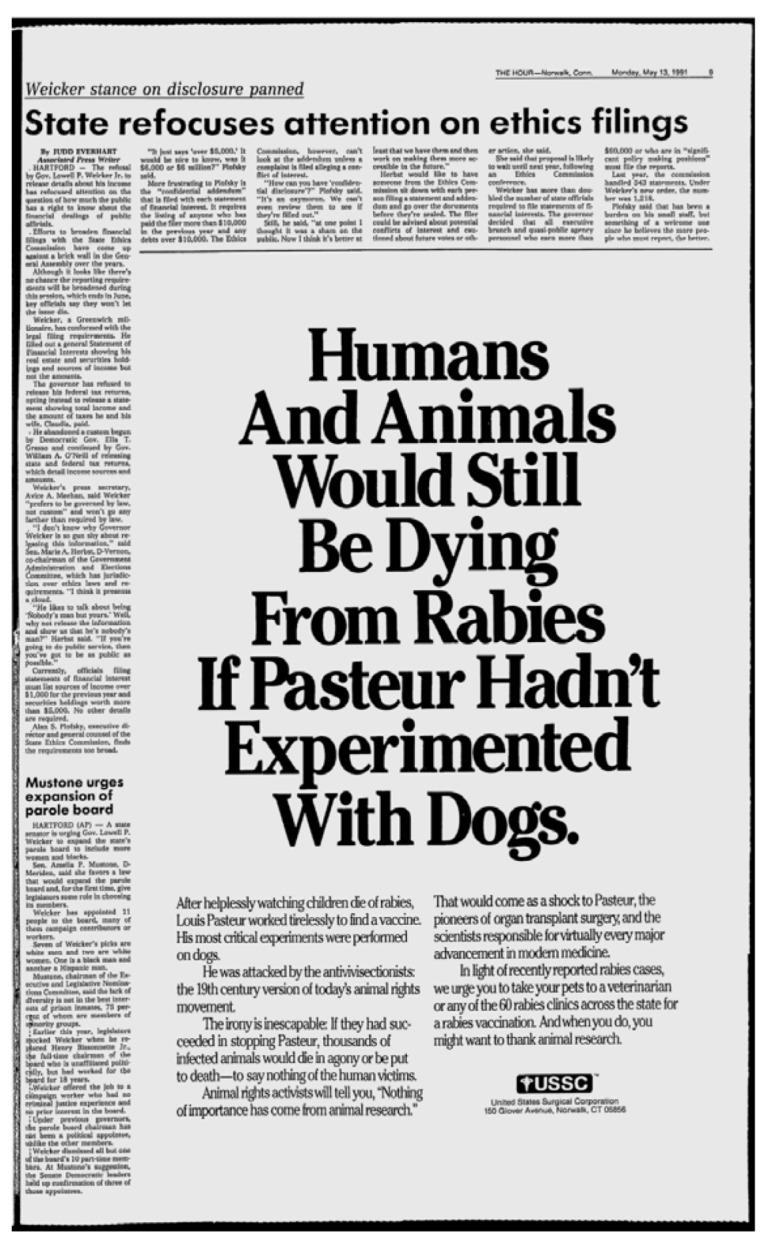
A large advertisement published in the 13 May 1991 edition of *The Hour* (p. 9), and part of a campaign in defense of animal research, sponsored by the United States Surgical Corporation. While the value of Pasteur’s work is undeniable, there is, however, no scientific grounding for the claim that only by experimenting on dogs would a vaccine for rabies have been developed, or that other animal models or even non-animal methods could not have been used to achieve this in over a century. These dramatic and biased portraits of animal research are now more uncommon, as an increasing number of scientists acknowledge the need to be more candid and open to objective discussion over the possibilities and limitations of animal research, and of the scientific process altogether.

From the impact of Singer’s and Regan’s works in society and the academic world, “animal ethics” would emerge as a whole new field of philosophical and bioethical studies, and, with it, new and diverse ethical views on animals—including on animal research—and of our duties towards them. However, despite the diversity of philosophical views on the use of animals, the public debate on animal research would become polarized between animal rights activists and animal research advocates. While the first would uphold an uncompromising abolitionist stand, one could also find on the opposite side several persons who did not regard animal research as a moral issue at all [167]. Furthermore, and despite the debate in the philosophical ground remained civilized—even between diametrically opposed perspectives, see, for example, [168]—in the “real world” the antagonism began to build up. In the 1970s, animal rights extremist groups began resorting to terrorist actions, thus becoming a serious problem for researchers and authorities in several Western countries still today. These actions more often consist of trespassing, raiding animal facilities and laboratories, damage to property, harassment and death threats to researchers, their families and neighbors. It has also sometimes escalated into kidnapping, car and mail bombings, arson of homes and research facilities, mailing of AIDS-contaminated razorblades, and violence against scientists and their family members [169,170]. These actions, which have been classified as unjustifiable and damaging to the animal rights cause by Tom Regan himself [171], made researchers close themselves within their community and avoid speaking publicly about their work [172,173,174], which in turn left pro-research advocacy to emotion-appealing campaigns, of the likes of the Foundation for Biomedical Research’s “Will I be alright, Doctor?” film [175], or the advertisement depicted in Figure 6. 

In spite of the emergence of the animal rights movement, animal research for biomedical purposes was—as it continues to be—seen as morally acceptable by the majority of the public [176,177]. It became, however, increasingly evident that animal suffering was morally and socially relevant, and that an ethical balance between the benefits brought about by biomedical progress and the due consideration to animal wellbeing should be sought. However, whilst antivivisection movements would only re-emerge in the late 1970s [5,178], the need for a more humane science had already been acknowledged and addressed within the scientific community as early as the 1950s. 

Following the first edition of the Universities Federation for Animal Welfare’s *Handbook on the Care and Management of Laboratory Animals *(1954), the organization’s founder Charles Hume commissioned that same year a general study on humane techniques in animal experimentation to zoologist and classicist (and overall polymath) William Russell (1925–2006) and microbiologist Rex Burch (1926–1996), under a project chaired by immunologist Peter Medawar (1915–1987), Nobel Prize laureate in 1960 [179,180,181]. From this work, Russell and Burch would develop the tenet of the “Three Rs”—*Replacement, Reduction, Refinement*—principles that would be extensively developed in their seminal book, *The Principles of Humane Experimental Technique *[182]. In this book, the authors argued “humane science” to be “best science,” going so far as to state that “If we are to use a criterion for choosing experiments to perform, the criterion of humanity is the best we could possibly invent.” *Replacement* was defined as “any scientific method employing non-sentient material [to] replace methods which use conscious living vertebrates”; *Reduction* as the lowering of “the number of animals used to obtain information of a given amount and precision”; and *Refinement* as the set of measures undertaken to “decrease in the incidence or severity of […] procedures applied to those animals which have to be used,” later including also the full optimization of the wellbeing of laboratory animals, also seen as a basic requirement for the quality of science [179]. They also challenged the commonly held belief that vertebrate animals—and mammals in particular—are always the most suitable models in biomedical research, a reasoning they called the *high-fidelity fallacy*. Despite receiving a warm welcome, Russell and Burch’s work would remain largely ignored well into the 1970s. In 1978, physiologist David Henry Smyth (1908–1979) would again bring the Three Rs to the light of day and encompass them under the concept of *alternatives* [67,183], which he defined as “all procedures which can completely replace the need for animal experiments, reduce the numbers of animals required, or diminish the amount of pain or distress suffered by animals in meeting the essential needs of man and other animals” [184]. More than a restatement of the Three Rs, this definition had the added value of placing onto researchers the burden of providing convincing evidence for the necessity of using animals [183], a particularly important statement from the then-president of the UK’s Research Defence Society. 

The Three Rs approach would provide an ethically and scientifically sound framework on which a reformist approach to the use of animals in biomedicine could be grounded. It would also set the stage for a more moderate advocacy of animal rights to appear: while remaining incompatible with an abolitionist animal rights perspective, this paradigm grants animals something like a right to protection from suffering, or at least certain suffering beyond a defined threshold [185], preserving the central idea that there are absolute and non-negotiable limits to what can be done to animals. This *welfarist* perspective stems from a utilitarian view that animals can be used as means to an end as long as their interests—as far as they can be ascertained—are taken into account, but also accepting that the lives and wellbeing of human beings must be granted greater consideration than animals’. Utilitarian philosopher Raymond G. Frey (1925–2012) offered a philosophical view compatible with the current paradigm, by acknowledging that what we do to animals matters morally, since animals’ sentience and ability to control their lives grants them moral standing and a rightful place in the “moral community.” However, when weighing the interests of humans against animals’ interests (or between animals, or humans), he held that the main question should not lie on one who has moral standing or not, or to which degree, but rather on whose life may be more valuable. In Frey’s view, the value of life “is a function of its quality, its quality of its richness, and its richness of its capacities and scope for enrichment.” Hence, as a result of their higher cognitive capabilities, human lives are typically richer than animal lives, being therefore generally more valuable [186]. 

A “welfarist–reformist” approach has been accepted as a compromise by some prominent animal rights advocates who, while maintaining the long-term goal of a full end to all animal experiments, believe that it is by successive short-term improvements of the *status quo* that their goal can be achieved; see [178,187,188]. This position—also endorsed by influential animal advocacy groups like the Humane Society of the United States, or the UK’s FRAME—has, however, been highly criticized by less compromising animal rights advocates, like Regan and Gary Francione (born 1954), who believe reformist attitudes validate and perpetuate the exploitation of animals [171,189,190]. 

**Figure 7:**
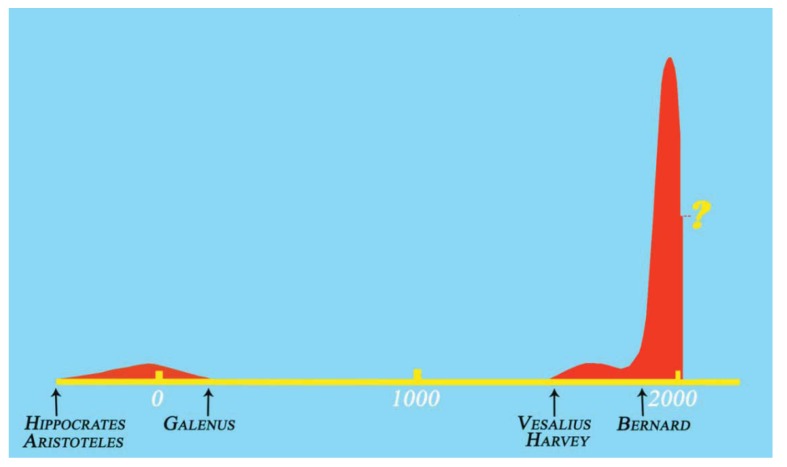
This schematic illustration (adapted with permission from an original by Professor Bert van Zupthen) attempts to describe trends in the use of animals for scientific purposes in the Western world across time. It depicts the emergence of the first vivisection studies by classical Greek physicians, the absence of animal-based research—along with most medical and scientific research—across the Middle Ages, its resurgence in the Renaissance onwards, and the rapid increase in animal studies following the rise of science-based physiology and medicine in the nineteenth century. The curves represented are nevertheless conjectural, as there are no reliable statistics on animal use for most of the period covered. Even nowadays it is hard to estimate trends in animal research, as data from several developed countries is insufficient (for instance, in the United States, rodents, fish and birds are not accounted for in the statistics). The available data, however, suggest that the number of animals used in research and testing in the Western world peaked in the 1970s, and decreased until the late 1990s, or early 2000s, to about half the number of 30 years earlier, and stabilizing in recent years. While many, if not most, researchers do not foresee an end to animal experiments in biomedicine, the European Commission has nevertheless set full replacement of animal experiments as an ultimate goal [204], and the Humane Society of the United States has the optimistic goal of full replacement by the year 2050 [192].

The 1980s and the 1990s would witness considerable progress in the development and acknowledgment of the Three Rs, to the satisfaction of William Russell and Rex Burch, who lived to see the “rediscovery” of their principles and the emergence of a whole new field of research inspired by their groundbreaking work [179,191]. As Peter Medawar had predicted in the 1960s, the number of animals used in research would peak in the 1970s and start to decline thereafter, although the number of biomedical papers has since then more than doubled [181,192,193,194,195,196]. This data is, however, limited to the Western world, as statistics on animal use in emerging countries such as India and China are unavailable [197], and there is no way to assess if (and, if so, to what extent) the decline in numbers of animals used in Western countries may be attributed to the outsourcing of animal experiments to these emerging countries. In recent years, the rise in the use of genetically modified animals has led to the stabilization of what would otherwise be a continuously downward trend [198,199] (Figure 7). 

In 1999, the Declaration of Bologna, signed in the 3rd World Congress on Alternatives and Animal Use in the Life Sciences, would reaffirm that “*humane science is a prerequisite for good science, and is best achieved in relation to laboratory animal procedures by the vigorous promotion and application of the Three Rs*” [200]. The Three Rs would also become the overarching principle of several legislative documents regulating animal use in science since the 1980s (including the latest European legislation [201]). Most recently, biomedical researchers in both industry and academia have also acknowledged the central importance of the Three Rs and the need for more transparency regarding animal use in biomedical research through the Basel Declaration [202,203]. More important, there are currently thousands of scientists devoted to the progress of animal welfare and development of alternatives to animal use in the life sciences. 

## 8. Conclusion

The historical controversy surrounding animal research is far from being settled. While the key arguments in this debate have not differed significantly since the rise of antivivisectionism in nineteenth-century England—and even before—we have since then moved a long way forward in regards to the protection of animals used in research and transparency regarding such use. While animal experiments have played a vital role in scientific and biomedical progress and are likely to continue to do so in the foreseeable future, it is nonetheless important to keep focusing on the continuous improvement of the wellbeing of laboratory animals, as well as further development of replacement alternatives for animal experiments. 
